# Hepatitis C Virus Transmission Clusters in Public Health and Correctional Settings, Wisconsin, USA, 2016–2017^1^

**DOI:** 10.3201/eid2702.202957

**Published:** 2021-02

**Authors:** Karli R. Hochstatter, Damien C. Tully, Karen A. Power, Ruth Koepke, Wajiha Z. Akhtar, Audrey F. Prieve, Thomas Whyte, David J. Bean, David W. Seal, Todd M. Allen, Ryan P. Westergaard

**Affiliations:** University, New York, New York, USA (K.R. Hochstatter);; University of Wisconsin School of Medicine and Public Health, Madison, Wisconsin, USA (K.R. Hochstatter, R. Koepke, W.Z. Akhtar, R.P. Westergaard);; London School of Hygiene and Tropical Medicine, London, UK (D.C. Tully);; Ragon Institute of MGH, MIT and Harvard, Cambridge, Massachusetts, USA (D.C. Tully, K.A. Power, D.J. Bean, T.M. Allen);; Wisconsin Department of Health Services, Madison (R. Koepke, R.P. Westergaard);; Wisconsin State Laboratory of Hygiene, Madison (A.F. Prieve, T. Whyte);; Tulane University School of Public Health and Tropical Medicine, New Orleans, Louisiana, USA (D.W. Seal)

**Keywords:** Hepatitis C virus, injection drug use, global hepatitis outbreak surveillance technology, molecular epidemiology, phylogenetics, viruses, transmission clusters, Wisconsin, United States, hepatitis

## Abstract

Ending the hepatitis C virus (HCV) epidemic requires stopping transmission among networks of persons who inject drugs. Identifying transmission networks by using genomic epidemiology may inform community responses that can quickly interrupt transmission. We retrospectively identified HCV RNA–positive specimens corresponding to 459 persons in settings that use the state laboratory, including correctional facilities and syringe services programs, in Wisconsin, USA, during 2016–2017. We conducted next-generation sequencing of HCV and analyzed it for phylogenetic linkage by using the Centers for Disease Control and Prevention Global Hepatitis Outbreak Surveillance Technology platform. Analysis showed that 126 persons were linked across 42 clusters. Phylogenetic clustering was higher in rural communities and associated with female sex and younger age among rural residents. These data highlight that HCV transmission could be reduced by expanding molecular-based surveillance strategies to rural communities affected by the opioid crisis.

Hepatitis C virus (HCV) infections have sharply increased in the United States, where an estimated 2.4 million persons are living with chronic infection (*1*). In 2013, ≈19,368 persons died of HCV-related complications, exceeding the number of deaths from all other nationally notifiable infectious diseases combined (*2*). During 2004–2014, prevalence of HCV increased by 2-fold, a direct result of the opioid epidemic and associated increases in the sharing of contaminated injection drug use equipment (*3*). Because the intersecting epidemics of opioid injection and infectious diseases are complex and dynamic, implementing community-specific comprehensive prevention services remains challenging.

Public health experts are increasingly using molecular-based surveillance techniques to identify and control emerging outbreaks (*4*–*7*). For example, the Centers for Disease Control and Prevention (CDC) has scaled up use of molecular HIV surveillance (*8*); however, the application of such programs for HCV surveillance has lagged. For molecular HCV surveillance and outbreak investigation, CDC developed a public health tool, Global Hepatitis Outbreak and Surveillance Technology (GHOST), which uses next-generation sequencing methods (*9*). GHOST integrates a suite of computational tools to accurately detect possible HCV transmission clusters from next-generation sequencing data in a simple fashion, regardless of the user’s level of expertise.

During 2016–2017, the rate of opioid overdose in Wisconsin increased by 109%, the steepest increase observed in any US state and nearly 3 times the average national increase over that period (*10*). This sharp increase in opioid use was accompanied by substantial increases in HCV incidence. During 2011–2015, an average of 2,955 new HCV diagnoses were reported annually; during the previous 5-year period, the average was 2,396. As a result of recent injection drug use, the rate of new PCR-confirmed HCV diagnoses among persons 15–29 years of age more than doubled during that period, from 40 to 87 cases/100,000 population (*11*).

In this study, we integrated public heath surveillance and molecular analyses with GHOST to identify putative HCV transmission clusters among persons most likely infected through injection drug use during a period of expanded HCV transmission (*12*). We also investigated the network characteristics among members of this high-risk group.

## Materials and Methods

### Study Setting and Population

All HCV-positive test results in Wisconsin are routinely reported to the Wisconsin Electronic Disease Surveillance System (WEDSS), a secure, Internet-based health information system used for the reporting, investigation, and surveillance of communicable diseases in Wisconsin. Blood samples collected for HCV RNA confirmatory testing at sites supported by the Wisconsin Division of Public Health (e.g., syringe services programs [SSPs], correctional facilities, local health departments, community-based organizations, and public health clinics) are processed at the Wisconsin State Laboratory of Hygiene and stored for 5 years. Approximately 15% of all HCV cases reported to WEDSS represent persons who underwent fee-exempt HCV RNA confirmatory testing through the state laboratory. The cohort of persons tested comprised primarily younger persons with a history of injection drug use, resulting from the types of organizations that submit test results to the state laboratory.

We identified persons confirmed to have an HCV RNA–positive sample analyzed at the state laboratory and reported to WEDSS for the first time during 2016–2017 by 2 methods. First, we identified 241 persons residing in rural catchment areas. Of the 72 counties in Wisconsin, 51 were included in the rural catchment area and selected on the basis of participation in an ongoing federally funded research program. These counties were classified as rural because they were served by 1 of the 6 rural offices of the statewide SSP. Second, to improve network completeness and compare the extent of clustering between rural and nonrural populations, we identified 2 additional cohorts: 54 persons residing in nonrural catchment areas and 164 residing in correctional facilities. Because resource limitations prevented data collection from all HCV-infected persons in nonrural catchment areas and correctional facilities, we included those who were considered likely to represent recent or acute infections because they either had acute HCV when reported to WEDSS or were 15–39 years of age at diagnosis with an HCV viral load >1,000,000 IU/L. The nonrural catchment area included the other 21 Wisconsin counties served by 1 of the 4 SSP urban offices, and the correctional cohort included those incarcerated in a state correctional setting (i.e., state prison) at the time of testing.

### Specimen Processing

Per standard protocol, the state laboratory stores serum remaining after completion of HCV antibody and RNA PCR testing at −80°C. Specimens corresponding to the HCV RNA–positive persons identified in WEDSS were retrieved and shipped to the Ragon Institute of MGH, MIT and Harvard (Cambridge, MA, USA) for virus sequencing.

### Nucleic Acid Extraction and PCR Amplification

RNA was isolated from 140 μL of plasma by using a QIAamp Viral RNA Mini Kit (QIAGEN, https://www.qiagen.com). A 1-step reverse transcription PCR (RT-PCR) was performed to amplify a 305-bp segment at the E1/E2 junction of the HCV genome (H77 positions 1301–1606), which contains the hypervariable region (HVR) 1 (*13*). This region was chosen for its high variability and its ability to reliably detect transmission events in outbreak settings (*14*). The first round of RT-PCR consisted of an Illumina adaptor-specific portion, a sample-specific barcode segment, and an HCV HVR–specific primer segment F1-GTGACTGGAGTTCAGACGTGTGCTCTTCCGATCT-NNNNNNNNNN-GGA-TAT-GAT-GAT-GAA-CTG-GT and R1-ACA-CTC-TTT-CCC-TAC-ACG-ACG-CTC-TTC-CGA-TCT-NNNNNNNNNN-ATG-TGC-CAG-CTG-CCG-TTG-GTG-T at a final concentration of 4 pM. Amplification conditions (SuperScript III One-Step RT-PCR System with Platinum Taq High Fidelity [ThermoFisher, https://www.thermofisher.com]) were cDNA synthesis for 30 min at 55°C followed by heat denaturation at 95°C for 2 min. PCR amplification conditions were 40 cycles of denaturation (94°C for 10 s), annealing (55°C for 10 s), and extension (68°C for 10 s) with a final extension at 68°C for 5 min. Amplified products were run on 1% agarose gel and either PCR purified with a QIAquick PCR Purification Kit (QIAGEN) or gel extracted and purified by using a PureLink Quick Gel Extraction Kit (Invitrogen, https://www.thermofisher.com). A second round of limited cycle PCR (94°C for 2 min, [94°C for 15 s; 55°C for 30 s; 68°C for 30 s] × 8 cycles, 68°C for 5 min) was performed to add barcode-specific indexes and sequencing-specific adapters and primers to each sample to allow for multiplexing as well as internal controls for cross-contamination. Negative controls were introduced at each stage, and all PCR procedures were performed under PCR clean room conditions by using established protocols. Indexed samples were purified by solid phase reversible immobilization (SPRI) 2 times at a bead-to-DNA ratio of 0.7× to remove excess primer dimer and short fragments that can interfere with the sequencing process.

### Deep Sequencing and Analysis

Resulting PCR amplicons were quantified by using a PicoGreen kit (Invitrogen) on a QuantiFluor ST fluorometer (Promega, https://www.promega.com), and the integrity of the fragment was evaluated by using a 2100 Bioanalyzer (Agilent, https://www.agilent.com). Samples were pooled and sequenced on an Illumina MiSeq platform (https://www.illumina.com) by using a 2 × 250–bp v2 Nano reagent kit. A sequence library consisted of 8–16 specimens, including 1 negative control for every 7 serum specimens. Paired-end reads were subject to stringent cleaning and quality control criteria as outlined previously (*15*–*17*). Duplicate reads were removed by using default settings with FastUniq version 1.1 (*18*) and quality trimmed by using Trimmomatic version 0.36 (*19*). Viral contigs were generated by using default settings with Vicuna version 1.1 (*20*), and a de novo consensus assembly was generated by using Viral Finishing and Annotation Toolkit (V-FAT) version 1.1 (https://www.broadinstitute.org/viral-genomics/v-fat). Read data are available from the National Center for Biotechnology Information Read Archive (https://www.ncbi.nlm.nih.gov) under BioProject accession no. PRJNA661611.

### Phylogenetic Reconstruction

We aligned the consensus sequences by using MEGA version 6.0 (*21*) and IQ-TREE version 1.6 (*22*). We then constructed a maximum-likelihood phylogenetic tree with 1,000 ultrafast bootstrap replicates (*23*).

### HCV Transmission Network Analyses

We uploaded Illumina paired-end reads to GHOST and subjected them to automatic quality control criteria. In brief, read pairs were filtered out if a read had >3 Ns (N indicates that software was not able to make a basecall for this base) or a length <185 bp. Each identifier on forward and reverse reads was examined, and the pair was discarded if either identifier was not an exact match to a given list of valid identifiers. We discarded pairs containing valid identifiers if they were not a constituent of the majority identifier tuple. If >11% of the read pairs contained valid identifiers that were not the majority tuple, we discarded the entire sample without further processing. Random subsampling of 5,000–20,000 read pairs was undertaken, and primer sequences were located in each read, allowing for a combined error total of <3. Read pairs were discarded when the primer could not be found. Remaining read pairs were then unified in a single error-corrected sequence, and only those sequences with a nonsense-free reading frame were collapsed into unique occurences with associated frequencies. Further methodologic details on quality filtering can be found elsewhere (*9*,*24*). We examined transmission links that represent the genetic similarity among virus populations from infected persons. For each case, we compared the intrahost populations between infected persons and calculated the genetic distance (defined as the Hamming distance) between their closest haplotypes. If the genetic distance is smaller than an empirically defined threshold of 3.77%, then samples are considered to be genetically related and indicate a transmission cluster (*14*). To further analyze each cluster’s genetic relationship, we built k-step networks of intrahost HCV HVR1 variants, as previously described (*9*).

### Data Collection

Variables routinely reported to WEDSS for HCV-positive persons include age, sex, race/ethnicity, HCV-positive antibody and RNA test date(s), testing site(s), and residential address. In addition, persons tested by the multisite SSP provide risk information per standard HCV testing procedures. Reporting of risk information is voluntary. When possible, local health department staff members gather risk information from the healthcare provider or directly from patients and enter it into WEDSS. Persons with HCV originally reported from state correctional facilities are not interviewed by local public health officials, and risk information for them is typically not available. When risk information was missing from WEDSS, we were unable to determine whether a patient answered “no” to a risk behavior or whether the data were missing. For persons who reported risk behaviors, we assessed whether they ever engaged in injection drug use, shared injection equipment, were men who have sex with men (MSM), or were ever incarcerated. Persons were considered ever incarcerated if any result for an HCV test conducted at a state correctional facility was reported to WEDSS or if the person reported (on risk information forms) having ever been incarcerated. Because availability of risk information depends on the type of facility where the person was tested, we present demographic characteristics and risk behaviors by type of testing facility: SSP, correctional facility, local health department, or other public venue. Other venues include a limited number of community health centers, public health clinics, community-based organizations, and safety net hospitals. Local jails also were considered other venues because only 2 persons were tested in jails and local jails are more representative of where the person resides, whereas persons may be placed in other facilities anywhere across the state regardless of their county of residence.

This study was approved by the University of Wisconsin Health Sciences Institutional Review Board, which granted a waiver of informed consent, and the Massachusetts General Hospital Institutional Review Board. Data Use Agreements and a Materials Transfer Agreement were established between the University of Wisconsin, Wisconsin Division of Public Health, the state laboratory, and the Ragon Institute of MGH, MIT and Harvard.

### Statistical Analyses

To compare clustering by demographics and risk behaviors, we conducted χ^2^, Fisher exact, Student *t*, and analysis of variance tests by using Stata SE 16 (StataCorp, https://www.stata.com). Because sampling techniques differed in rural and nonrural catchment areas and the characteristics assessed were strongly determined by which catchment area persons were in, and because persons tested in correctional facilities could come from either rural or nonrural areas of the state, we compared persons who clustered with those who did not cluster, stratified by 3 groups based on testing location: the rural catchment area, the nonrural catchment area, and correctional facilities. We also compared characteristics between rural catchment area–only clusters, nonrural catchment area–only clusters, corrections-only clusters, and clusters that contained persons from >1 group. Statistical significance was determined by using α<0.05.

## Results

### Study Sample

During 2016–2017, a total of 459 persons tested by the Wisconsin State Laboratory of Hygiene were HCV RNA positive for the first time. For those 459 persons, sufficient (>200 μL) residual serum was stored to enable virus sequencing for 424 (92.4%). Of these, virus was successfully amplified, sequenced, and passed GHOST quality control metrics for 379 (89.4%) samples. Among the samples that failed, 23 (5.4%) failed PCR and 22 (5.2%) failed GHOST quality control metrics. After quality control, the median number of error-corrected reads/person was 16,740 (interquartile range 13,302–18,262) and the median number of haplotypes was 3,322 (interquartile range 2,479–4,345).

### Patient Demographic Characteristics and Risk Behaviors

Among the 379 persons whose specimens were successfully analyzed by GHOST, positive HCV results were first obtained at an SSP for 119 (31.4%), a correctional facility for 154 (40.6%), a local health department for 38 (10.0%), and other settings for 68 (17.9%) (Table 1). The study population was primarily non-Hispanic white (83.9%), 18–39 years of age (90.8%), and male (75.5%). Self-reported injection drug use was documented for 177 (46.7%) persons. Of these, 145 (81.9%) self-reported having ever shared injection equipment. MSM status was reported by 8 (2.1%) persons. Most of the study population (335 [88.4%]) had been incarcerated; 154 received their first HCV-positive test result while at a correctional facility, and 180 reported a history of incarceration.

**Table 1:** Demographics and risk factor information, by type of testing facility, for all 379 persons tested for HCV while in public health or correctional settings, Wisconsin, USA, 2016–2017*

Variable	Overall no. (%)	Setting of first HCV-positive test result, no. (%)			
		Syringe services program	Corrections setting	Local health department	Other
Total	379 (100)	119 (31.4)	154(40. 6)	38 (10.0)	68 (17.9)
Year first reported to WEDSS					
2016	222 (58.6)	68 (57.1)	87 (56.5)	22 (57.9)	45 (66.2)
2017	157 (41.4)	51 (42.9)	67 (43.5)	16 (42.1)	23 (33.8)
Age, y					
18–29	190 (50.1)	57 (47.9)	77 (50.0)	19 (50.0)	37 (54.4)
30–39	154 (40.6)	39 (32.8)	75 (48.7)	16 (42.1)	24 (35.3)
>40	35 (9.2)	23 (19.3)	2 (1.3)	3 (7.9)	7 (10.3)
Race/ethnicity					
Non-Hispanic white	318 (83.9)	103 (86.6)	127 (82.5)	34 (89.5)	54 (79.4)
Hispanic or Latino	18 (4.8)	4 (3.4)	7 (4.6)	2 (5.3)	5 (7.4)
American Indian or Alaska Native	21 (5.5)	7 (5.9)	9 (5.8)	1 (2.6)	4 (5.9)
Asian	2 (0.5)	0	1 (0.7)	0	1 (1.5)
Non-Hispanic black or African American	12 (3.2)	4 (3.4)	5 (3.3)	1 (2.6)	2 (2.9)
Other/unknown	8 (2.1)	1 (0.8)	5 (3.3)	0	2 (2.9)
Sex					
F	93 (24.5)	44 (36.9)	14 (9.1)	13 (34.2)	22 (32.3)
M	286 (75.5)	75 (63.0)	14 (90.9)	25 (65.8)	46 (67.7)
Ever injected drugs					
Yes	177 (46.7)	98 (82.4)	16 (10.4)	32 (84.2)	31 (45.6)
No or unknown	202 (53.3)	21 (17.7)	138 (89.6)	6 (15.8)	37 (54.4)
Ever shared works					
Yes	145 (38.3)	88 (74.0)	10 (6.5)	29 (76.3)	18 (26.5)
No, unknown, or NA	234 (61.7)	31 (26.1)	144 (93.5)	9 (23.7)	50 (73.5)
MSM					
Yes	8 (2.1)	4 (3.4)	0	2 (5.3)	2 (2.9)
No or unknown	371 (97.9)	115 (96.6)	154 (100)	36 (94.7)	66 (97.1)
Ever incarcerated					
Yes	335 (88.4)	92 (77.3)	154 (100)	34 (89.5)	54 (79.7)
No or unknown	44 (11.6)	27 (22.7)	0	4 (10.5)	14 (20.6)

*HCV, hepatitis C virus; MSM, men who have sex with men; NA, not applicable; WEDSS, Wisconsin Electronic Disease Surveillance System.

Among the 379 persons, 171 (45.1%) resided in the rural catchment area, of which 67 (39.2%) clustered; 54 (14.3%) resided in the nonrural catchment area, of which 14 (25.9%) clustered; and 154 (40.6%) resided in correctional facilities, of which 45 (29.2%) clustered. Among the 171 persons in the rural catchment area, women were significantly more likely to cluster (49%) than men (33%) (p = 0.04), and persons who clustered were significantly younger (mean age 28.7 years) than persons who did not cluster (mean age 34.1 years) (p = 0.0001). For in the nonrural catchment area or corrections groups, we found no statistically significant differences between those who clustered and those who did not cluster.

### Phylogenetic Analysis

**Figure 1:**
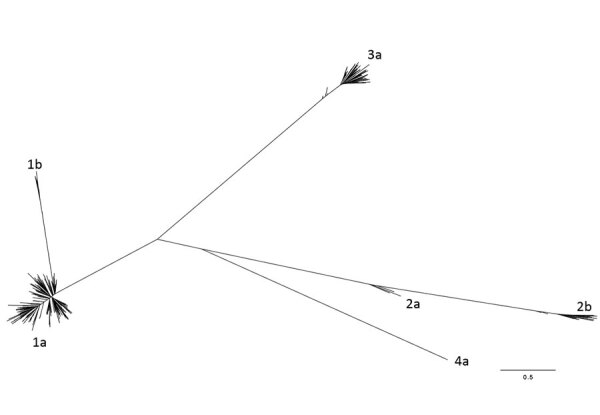
Maximum-likelihood phylogenetic tree of hepatitis C virus hypervariable region 1 consensus sequences from samples from 379 persons in public health and corrections settings, Wisconsin, USA, 2016–2017. The breadth of genetic diversity is shown, and genotypes are labeled. Scale bar indicates nucleotide subsitutions per site.

We genetically characterized HCV strains by using HVR1 consensus sequences derived from isolates from all 379 persons. Phylogenetic analysis demonstrated a predominance of genotypes 1a (n = 255, 67.3%) and 3a (n = 88, 23.2%), followed by 2b (n = 22, 5.8%), 1b (n = 9, 2.4%), 2a (n = 4, 1.1%), and 4a (n = 1, 0.3%) (Figure 1).

### HCV Transmission Linkages

**Figure 2:**
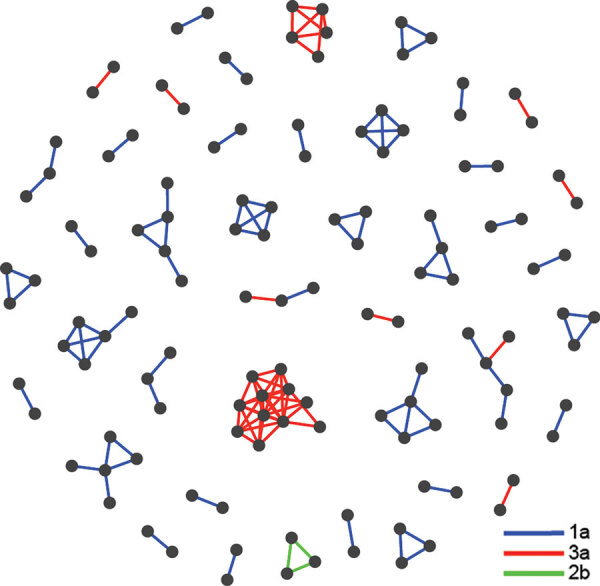
Hepatitis C virus (HCV) transmission network among persons in public health and corrections settings, Wisconsin, USA, 2016–2017, showing 42 clusters identified by Global Hepatitis Outbreak and Surveillance Technology (GHOST). Each node represents an HCV-infected person for whom HCV sequence data were generated. A transmission link is denoted as a line connecting persons where the minimal Hamming distance between sequences is smaller than the previously validated genetic threshold of 3.77%. Lines connecting clusters are colored according to genotype.

GHOST detected 42 clusters comprising 126 persons for an overall clustering rate of 33% (Figure 2). Cluster sizes ranged from 2 to 11 persons. Transmission networks were composed of mostly dyads (n = 23, 54.8%), followed by groups of 3 (n = 9, 21.4%), 4 (n = 3, 7.1%), and 5 (n = 6, 14.3%). The largest cluster involved 11 persons, all infected with genotype 3a. Among those 11 persons, 5 received their first HCV-positive test result from the same local health department and 3 from the same SSP. Also among those 11 persons, evidence of past injection drug use was available for 7 persons, 8 were male, and all 11 were non-Hispanic white with a history of incarceration.

Among the 42 clusters identified, none comprised only persons residing in the nonrural catchment area, 12 comprised only persons residing in the rural catchment area (n = 34), 7 comprised only persons from corrections settings (n = 15), and 23 comprised persons from >1 group (n = 77). Rural catchment area–only clusters were more likely to comprise a higher percentage of women (47.1%) compared with 6.7% of corrections-only clusters and 27.3% of mixed clusters; this finding probably results from the higher incarceration rate among men. We found no other significant differences in demographics between rural-only, corrections-only, and mixed clusters. We were unable to compare risk behaviors between these cluster types because limited risk behavior data were available for corrections settings–only clusters, there were no urban-only clusters, and mixed clusters comprised many persons from corrections settings. 

### Intrahost Genetic Variation within Transmission Clusters

**Figure 3:**
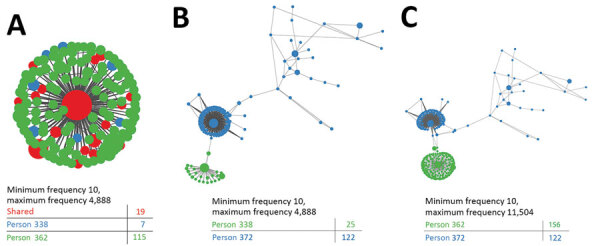
Hepatitis C virus (HCV) transmission network among persons in public health and corrections settings, Wisconsin, USA, 2016–2017, showing intrahost genetic heterogeneity within 1 representative transmission cluster. K-step network contains all possible minimum spanning trees and enables efficient visualization of genetic relatedness among all intrahost hypervariable region 1 (HVR1) variants for persons 338 and 362 (A), persons 338 and 372 (B), and persons 362 and 372 (B). Each node represents an HCV sequence, and the color of the node corresponds to the sample of origin: red, variant found in both samples; green, variant found only in the first sample; blue, variant found only in the second sample. Node size is based on frequency of the HVR1 variant, and edge length is proportional to the modified Hamming distance (does not count positions with insertions or deletions as differences).

**Figure 4:**
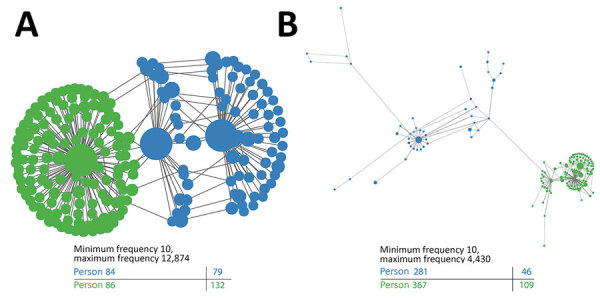
Intrahost genetic variation of representative transmission clusters of hepatitis C virus (HCV) among persons in public health and corrections settings, Wisconsin, USA, 2016–2017, highlighting the genetic relatedness of distinct variants. K-step network contains all possible minimum spanning trees and enables efficient visualization of genetic relatedness among all intrahost hypervariable region 1 (HVR1) variants for persons 84 and 86 (A) and persons 281 and 367 (B). Each node represents an HCV sequence. Color of the node corresponds to the sample of origin: green, found only in the first sample; blue, found only in the second sample. The node size is based on frequency of the HVR1 variant, and edge length is proportional to the modified Hamming distance (does not count positions with insertions or deletions as differences).

GHOST analysis of the intrahost HVR1 variants revealed that 5 (1.3%) of the 379 persons were infected with multiple strains of HCV (Table 2). To further describe the nature of HCV transmission across clusters, we examined the population structure of HVR1 variants to address whether the same virus variant was shared among HCV-infected persons as previously described (*14*). Although it is not possible to illustrate the k-step networks for each sample, we highlight representative examples of clusters. One cluster comprised 3 persons (nos. 372, 338, and 362), and persons 338 and 362 harbored little viral intrahost genetic variation and shared 19 viral variants (modified Hamming distance = 0) (Figure 3, panel A). The third person, no. 372, was infected with many virus variants with a single subpopulation that is genetically similar to variants found in persons 338 (Figure 3, panel B) and 362 (Figure 3, panel C). Another representative cluster comprised a simple dyad of persons (nos. 84 and 86) who shared a virus variant (modified Hamming distance = 0.37) with only a minor difference between each (Figure 4, panel A). In contrast, the cluster of persons 281 and 367 shared a more distantly related variant (modified Hamming distance = 3.18) (Figure 4, panel B).

**Table 2:** Characteristics of samples with mixed hepatitis C virus genotypes, collected from persons in public health or correctional settings, Wisconsin, USA, 2016–2017

Sample/person no.	Major genotype (%)	Minor genotype (%)	Risk factor*
116	1a (96.75)	2a (3.25)	Unknown
63	1a (99.38)	3a (0.62)	Ever injected drugs, ever shared works
318	1a (96.18)	3a (3.82)	Unknown
338	1a (99.72)	6f (0.28)	Ever injected drugs
306	1a (98.57)	2b (0.59)	Ever injected drugs, ever shared works

*When this evidence is available, the person can be classified as man who has sex with men, ever injected drugs, or ever shared works.

## Discussion

This study demonstrates the ability to link statewide public health surveillance to HCV transmission clusters identified by GHOST. The 33% rate of clustering that we found among key affected populations in Wisconsin is comparable to that found in Vancouver, British Columbia, Canada (where 31% of persons who inject drugs [PWIDs] cluster), and Baltimore, Maryland, USA (where 46% of PWIDs cluster) (*25*,*26*). However, those prior studies included only PWIDs from their respective metropolitan areas. Our study included both urban and rural populations. We found a higher rate of clustering in the rural catchment area, and rural persons who clustered were younger, a finding that aligns with the literature describing the particular burden of HCV on young persons in rural communities (*27*,*28*). Moreover, these data highlight that the increasing rurality of opioid injection and HCV transmission among young PWIDs could be better supported by the expansion of molecular-based surveillance strategies to reduce transmission. The availability of transmission networks would enable targeting of the underlying contact network structure such that persons who are highly central within a network contribute much more to infection than those on the periphery. This type of network-based disruption strategy has been shown to reduce incidence more than randomly targeted prevention strategies (*29*).

Use of molecular epidemiologic methods to investigate transmission of infectious diseases addresses many limitations of traditional contact tracing, for which data collection is often time-intensive and results may be subject to recall and social desirability biases. Contact tracing among persons who engage in illegal activity is especially challenging because these persons are often reluctant to disclose injecting behaviors or name injecting partners because of stigma or fear of criminal repercussions (*30*). Therefore, identifying transmission linkages with GHOST can support more targeted contact tracing strategies. Unfortunately, contact tracing was not performed among our study population, precluding further analyses. Because modeling studies have demonstrated that HCV can be eliminated through scaling up and targeting treatment (*31**,**32*), a concept known as treatment as prevention, often used in HIV research (*33**,**34*), conducting routine molecular surveillance may also advance HCV prevention efforts by facilitating efficient allocation of limited resources to target and treat members in clusters.

The first limitation of our study is that HCV testing and surveillance challenges make identifying a complete cohort of HCV-infected PWIDs difficult. CDC estimates that approximately half of all HCV-infected persons are unaware of their infection status (*35*). Persons not included in our analysis include those who were never tested, were tested outside of Wisconsin, or were tested in other settings (e.g., primary care) that use commercial or hospital-based laboratories for HCV testing. Accordingly, the population studied is not fully representative of the Wisconsin general population. However, our results do provide a credible picture of the HCV epidemic across public health and correctional settings throughout rural and urban Wisconsin. Second, the association we found between clustering and younger age among rural residents could result from having sampled a larger number of younger persons. Third, risk-behavior data are missing for a large proportion of the sample because few agencies routinely collect and report these data. Data may also be missing because persons may choose to not disclose potentially stigmatizing drug use and sexual behaviors, particularly in settings such as correctional facilities ,where persons may fear further punishment. Fourth, we were unable to determine from which catchment area persons in corrections facilities orginated. Last, phylogenetic clustering alone cannot directly assert whether transmission has occurred between persons (*36*). 

In conclusion, our findings provide a snapshot of the HCV epidemic throughout Wisconsin during 2016–2017. They illustrate the need to especially direct resources to rural communities affected by the opioid crisis.
